# Rigid anterior plating for open transverse sternal fracture traversing previous sternotomy: a case report

**DOI:** 10.1186/s13019-026-04467-1

**Published:** 2026-07-09

**Authors:** Vinh Duc An Bui, Dat Nhu Nguyen, Phuoc Nguyen Le, Binh Thanh Tran

**Affiliations:** 1 Department of Thoracic and Cardiovascular Surgery, Hue Central Hospital, Hue, Vietnam; 2 Department of Thoracic Surgery, 103 Military Hospital, 261 Phung Hung Street, Ha Dong Ward, Hanoi, Vietnam

**Keywords:** Sternal fracture, Rigid plate fixation, Previous sternotomy, Retrosternal adhesions, Chest wall trauma, Case report

## Abstract

**Background:**

Traumatic sternal fractures account for 0.33–8% of blunt chest trauma admissions and are usually managed conservatively. However, displaced or unstable fractures may require surgical fixation. Fractures traversing a previous median sternotomy are exceptionally rare and present unique challenges due to retrosternal adhesions and the risk of injury to cardiac structures during posterior dissection. No prior reports have specifically described anterior-only rigid plate fixation for traumatic sternal fracture crossing a previous sternotomy in a patient with mechanical heart valves. We present such a case and discuss the surgical rationale and outcome.

**Case presentation:**

A 64-year-old man with a history of mechanical mitral and aortic valve replacement 27 years previously presented after a motorcycle collision, sustaining an overlying parasternal skin laceration and a completely displaced, open transverse sternal fracture at the third intercostal space, crossing the previous sternotomy line. Intraoperative exploration confirmed direct communication between the parasternal laceration and the fracture site, establishing the diagnosis of a true open sternal fracture (Gustilo–Anderson type II). Associated injuries included multiple non-displaced right-sided rib fractures, pulmonary contusion, and bilateral hemothoraces. Given the fracture instability, wound contamination risk, and the need for anticoagulation, urgent surgical stabilization was performed. Two parallel one-third tubular titanium plates with monocortical locking screws were applied via anterior-only approach, deliberately avoiding retrosternal dissection to prevent injury to cardiac structures adherent to the posterior sternum. The postoperative course was uneventful. At 3-month follow-up, computed tomography confirmed correct alignment with a smooth posterior contour and no hardware protrusion into the mediastinum.

**Conclusions:**

Anterior-only rigid plate fixation is a safe and effective strategy for traumatic sternal fractures crossing a previous sternotomy, avoiding the hazards of retrosternal dissection while providing adequate biomechanical stability. Preoperative review of prior cardiac surgical history is essential when planning sternal fracture fixation.

**Supplementary Information:**

The online version contains supplementary material available at https://doi.org/10.1186/s13019-026-04467-1.

## Background

Sternal fractures are uncommon injuries, accounting for approximately 0.33–8% of trauma admissions [1]. Most result from high-energy blunt chest trauma, predominantly motor vehicle crashes and motorcycle collisions. The majority of sternal fractures can be managed conservatively with adequate analgesia and respiratory support, as they typically heal uneventfully without surgical intervention.

However, displaced or unstable fractures associated with persistent pain, respiratory compromise, open wounds, or failure of conservative management require surgical stabilization [2]. Sternal instability can directly impair ventilation by impairing chest wall mechanics, leading to atelectasis, hypoventilation, and pneumonia, which are well-recognized indications for surgical stabilization in chest wall trauma [1, 2]. Various fixation techniques have been described, including wire cerclage and rigid plate systems. Randomized trials have demonstrated superior biomechanical stability and union rates with rigid plate fixation compared with wire cerclage [3, 4]. The Arbeitsgemeinschaft für Osteosynthesefragen / Orthopaedic Trauma Association (AO/OTA) classification provides a standardized framework for describing sternal fractures, though it does not account for fractures complicated by prior sternal surgery [5].

Traumatic sternal fractures traversing a previous median sternotomy pose distinct surgical challenges. After cardiac surgery, dense retrosternal adhesions frequently develop between the pericardium, vital cardiovascular structures (specifically the innominate vein, aorta, and right ventricle), and the posterior sternal surface [6, 7]. The risk of catastrophic injury to cardiac structures during redo sternotomy is well-documented, reported at 1–5% even in planned cardiac reoperations [6]. In patients with mechanical heart valve prostheses requiring lifelong anticoagulation, this risk is further compounded by the bleeding diathesis.

To our knowledge, no prior case report has specifically described the management of a traumatic sternal fracture crossing a previous sternotomy using an anterior-only rigid plate fixation strategy. We report such a case and discuss the surgical decision-making and outcome.

## Case presentation

A 64-year-old man was admitted to our hospital following a motorcycle collision with direct blunt impact to the anterior chest. His past surgical history was significant for mechanical mitral and aortic valve replacement 27 years previously, requiring continual vitamin K antagonist therapy.

**Fig. 1:**
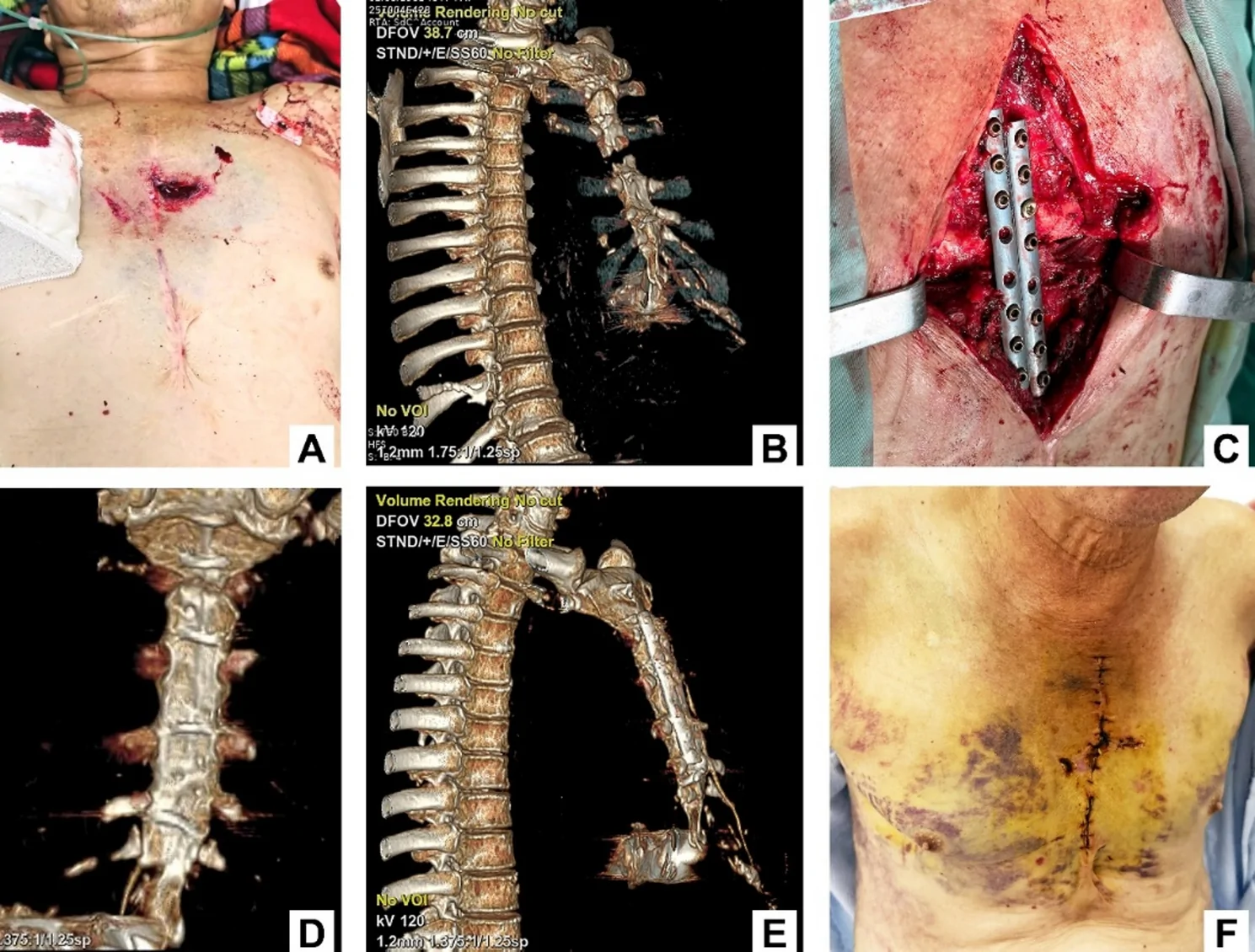
Imaging and operative findings of the transverse sternal fracture crossing the previous sternotomy. **A**, Initial presentation showing an open parasternal wound. **B**, Preoperative three-dimensional CT demonstrating a completely displaced transverse fracture at the third intercostal space crossing the old sternotomy line. **C**, Intraoperative view after anatomic reduction and fixation using two parallel one-third tubular titanium plates secured with monocortical screws. **D**, Postoperative CT posterior view showing a smooth posterior contour without screw protrusion. **E**, Oblique three-dimensional CT reconstruction confirming correct alignment. **F**, Healed incision at discharge with restored sternal stability

On examination, the patient was hemodynamically stable with a 2 cm open laceration adjacent to the left side of the old midline sternotomy scar and visible paradoxical chest wall motion (Supplementary Video S1). On arrival he was conscious and alert, with mild dyspnoea. Vital signs were a respiratory rate of 20 breaths/min, oxygen saturation of 94% on a nasal cannula, heart rate of 80 beats/min, and blood pressure of 100/70 mmHg. Subcutaneous fat globules were seen draining through the parasternal wound. The patient reported severe pain with inspiration and exhibited shallow breathing, suggesting impaired ventilatory mechanics secondary to chest wall instability. The open wound was provisionally noted to be in close proximity to the fracture site; intraoperative exploration subsequently confirmed direct continuity between the parasternal laceration and the fracture, confirming an open fracture. Based on the moderate soft-tissue laceration overlying the fracture, the injury was classified as Gustilo–Anderson type II. Multislice computed tomography (CT) with three-dimensional reconstruction revealed a completely displaced transverse fracture at the third intercostal space, crossing the previous sternotomy line (Fig. 1A, B). Associated findings included multiple non-displaced right-sided rib fractures (ribs 3–5), pulmonary contusion, and bilateral hemothoraces. The rib fractures were minimally displaced and did not contribute to overall chest-wall instability, and the intercostal spaces adjacent to the sternal fracture were intact, with no soft-tissue disruption. Both clinical examination and CT excluded any associated thoracic spine fracture or distraction injury. Preoperative laboratory investigations showed a high-sensitivity Troponin T of 0.04 ng/ml and an international normalized ratio of 2.16. Electrocardiogram revealed no abnormalities. Transthoracic echocardiography demonstrated minimal pericardial effusion without evidence of prosthetic valve dysfunction.

Given the completely displaced fracture with paradoxical chest wall motion, the overlying open laceration with contamination risk, the impaired ventilatory mechanics, and the need for early resumption of therapeutic anticoagulation for double mechanical valves, urgent surgical stabilization was deemed necessary. Alternative management strategies, including temporary internal stabilization with positive-pressure mechanical ventilation, initial wound management with negative-pressure wound therapy, and delayed surgical fixation, were considered. However, these were deemed inferior in our patient because the gross fracture displacement with paradoxical motion would not be adequately controlled by ventilation alone, and delay risked progressive wound contamination and deteriorating respiratory function. Early definitive fixation allowed effective pain control, early mobilization, and prompt resumption of anticoagulation. Surgery was performed approximately three and a half hours after admission. During this interval the patient’s respiratory distress remained mild and was managed with supplemental oxygen and intravenous analgesia, with endotracheal intubation reserved for induction of anaesthesia. The supratherapeutic international normalized ratio was corrected preoperatively with intravenous vitamin K and fresh frozen plasma to achieve adequate haemostasis before incision.

A midline incision along the previous surgical scar was performed. Presternal dissection confirmed the CT findings of a completely displaced transverse fracture and intact cerclage wires from the prior cardiac surgery. The longitudinal midline sternotomy was stable, with the cerclage wires intact and well incorporated, and the only mechanical instability was the transverse traumatic fracture. Retrosternal dissection was deliberately avoided due to anticipated dense adhesions after 27 years of cardiac surgery, thereby preventing potential injury to cardiac structures adherent to the posterior sternum.

The traumatic laceration and surrounding devitalized tissue were widely excised until healthy bleeding margins were obtained. The wound was irrigated copiously with warm saline and diluted povidone–iodine solution prior to fixation. Empiric intravenous prophylactic antibiotics were initiated on admission and continued for 5 days postoperatively. The fracture edges were debrided to healthy bleeding bone and anatomic reduction was achieved. Two parallel one-third tubular titanium plates with monocortical locking screws were applied for fracture fixation (Fig. 1C). Plates were placed longitudinally on either side of the midline, spanning intact sternal segments above and below the fracture, with at least three screws engaged per fragment to achieve balanced load distribution. Preoperative CT measurements of sternal thickness at each planned screw site were used to select screw lengths 2–3 mm shorter than the minimum local thickness, ensuring strictly monocortical purchase. Depth-limited drilling with a calibrated drill stop was used to avoid inadvertent bicortical penetration. Anterior monocortical screws were specifically chosen to avoid mediastinal penetration. Bilateral chest tubes were placed for the associated hemothoraces.

The postoperative course was uneventful over a 6-day hospital stay. Following fixation, respiratory excursions normalized and the patient was successfully weaned from supplemental oxygen on postoperative day 2. At discharge, clinical examination demonstrated proper wound healing (Fig. 1F) and restored sternal stability. Follow-up CT at 3 months demonstrated correct alignment, a smooth posterior sternal contour, and no screw protrusion into the mediastinum (Fig. 1D, E). The patient returned to normal daily activities without chest wall pain.

## Discussion

This case represents, to our knowledge, the first reported instance of a traumatic sternal fracture crossing a previous median sternotomy managed with anterior-only rigid plate fixation. While sternal fixation techniques are well-established for post-sternotomy dehiscence and primary traumatic fractures [8, 9], the combination of a high-energy traumatic fracture, open wound, retained cardiac prostheses, and dense retrosternal adhesions presented a unique clinical scenario not previously addressed in the literature.

The most critical surgical decision was the deliberate avoidance of retrosternal dissection. After cardiac surgery, pericardial and great vessel adhesions to the posterior sternal surface develop progressively [6]. Recent studies using preoperative computed tomography and cardiac magnetic resonance imaging have demonstrated that retrosternal adhesions are present in the majority of patients after prior sternotomy, with the risk of cardiovascular injury during re-entry reported to range from 1% up to 9% [6, 7]. After 27 years, these adhesions are certain to be tenacious. Additionally, the patient’s ongoing anticoagulation for mechanical valve prostheses significantly increased the bleeding risk associated with any posterior dissection. The anterior-only approach eliminated these hazards entirely.

Wire cerclage was considered inadequate for several reasons. Beyond the technical necessity of posterior passage, which we specifically aimed to avoid, wires provide limited resistance to shear forces generated by transverse fractures. Randomized multicenter trials have demonstrated that rigid plate fixation offers superior biomechanical stability compared with wire cerclage. Raman et al. reported significantly better CT healing scores at three months with rigid plating compared with wires (1.7 ± 1.1 vs. 0.9 ± 0.8, *p* = 0.003) [4]. Allen et al. confirmed these findings, reporting higher rates of complete sternal union at six months (80% vs. 67%, *p* = 0.03) [3]. We selected one-third tubular titanium plates with monocortical locking screws to provide rigid fixation while ensuring that no hardware penetrated the mediastinal space. The use of titanium is preferred in potentially contaminated fields due to its inert biocompatibility [8]. Depth-limited drilling, as described by Schulz-Drost et al., provides additional safety during screw placement [2], and was used in this case in conjunction with CT-based pre-measurement to select screw lengths with an adequate safety margin.

In the setting of an open fracture with a contaminated wound field, implant infection is a significant concern. We addressed this by wide debridement of the laceration and surrounding devitalized tissue, copious wound irrigation prior to implant placement, the use of titanium plates for their biocompatibility and lower infection risk in contaminated fields [8], and a short course of perioperative intravenous antibiotic prophylaxis tailored to cover skin flora and extended to 5 days because of the open fracture and need to resume anticoagulation early, which is consistent with recommendations for high-risk fracture-related infection prophylaxis in trauma patients [10]. Closed suction drainage was used to minimize hematoma formation, which can predispose to late implant infection. No signs of wound infection or hardware complication were observed at 3-month follow-up.

Alternative management approaches for complex sternal injuries include temporary stabilization with positive-pressure ventilation, adjunctive closed-incision negative-pressure wound therapy to reduce sternal wound complications in high-risk patients [11], and conservative management with analgesics. However, a meta-analysis of randomized trials of surgical stabilization for flail chest demonstrated that operative fixation significantly reduced the duration of mechanical ventilation, pneumonia, and intensive care unit and hospital length of stay compared with nonoperative management [12]. In our patient, these conservative or staged approaches were considered inadequate given the gross mechanical instability, open wound, impaired ventilatory mechanics, and the urgent need to resume anticoagulation. Early definitive fixation therefore provided simultaneous fracture stabilization, wound control, and early mobilization.

Our surgical approach and outcome are consistent with published sternal plating series. Ahmed and Nady reported excellent pain reduction and early mobilization following titanium plate fixation for five patients with traumatic sternal fractures [9]. Voss et al. demonstrated clinical stability in all cases with complete or near-complete bone consolidation in over 60% at a median follow-up of 1.4 years [13]. Byun et al. compared T-shaped and longitudinal plates for sternal fracture fixation, finding that T-shaped plates provided superior fixation stability [14]. Our choice of two parallel longitudinal plates provided bilateral stabilization of the transverse fracture, a strategy supported by the biomechanical principle that dual plates offer greater resistance to bending and torsional forces than a single plate.

In our patient the previous longitudinal sternotomy was intraoperatively confirmed to be stable, so the only unstable plane was the transverse fracture, which the anterior-only construct addressed directly. A more complex situation would arise if transverse instability coexisted with dehiscence of the longitudinal midline. In such a combined two-plane injury, a single pair of paramedian longitudinal plates would be insufficient, as each plane requires separate neutralization. Reported options include reinforcing midline wire cross-connection with additional transverse plates spanning rib to rib, as described by Voss et al. for complex dehiscence, or using dedicated multi-arm plates such as H-shaped or star-shaped configurations that bridge both components simultaneously [13]. These constructs are not universally available and add operative complexity, and when retrosternal adhesions preclude posterior passage, a purely anterior combination of longitudinal and transverse low-profile plates with monocortical, depth-limited screw fixation represents a reasonable compromise [2].

The open wound in this blunt mechanism is best explained by local biomechanics rather than direct external penetration. The frontal impact drove the displaced sternal fragments outward against the thin presternal soft tissue overlying the old, relatively avascular scar, and the sharp bone edge perforated this attenuated skin from within, producing an open fracture from the inside out. This is consistent with the intraoperative finding of direct continuity between the laceration and the fracture and with the fat globules draining through the wound on admission. As the associated rib fractures were few and minimally displaced and did not create a flail segment, chest-wall stability was governed almost entirely by the sternal injury, and rib fixation was not required. In patients with multiple displaced rib fractures or a lateral flail segment, concomitant rib osteosynthesis should be considered to restore overall chest-wall stability [1].

This case underscores anterior-only rigid plating as a practical and safe strategy for complex sternal fractures traversing a previous sternotomy, completely circumventing the re-entry risks traditionally associated with posterior dissection. In patients with prior sternotomy and anticipated dense retrosternal adhesions, this approach provides sufficient mechanical stability while avoiding life-threatening cardiovascular injury.

This report has inherent limitations common to case reports. It describes a single patient, and the anterior-only approach may not be generalizable to all fracture patterns or clinical scenarios. The follow-up period of 3 months, while sufficient to confirm fracture alignment and early healing, does not capture long-term outcomes such as late hardware complications or the need for implant removal. Biomechanical testing of anterior-only monocortical fixation versus bicortical or combined anterior–posterior constructs was not performed. Prospective studies comparing fixation strategies in patients with prior sternotomy would be valuable, though the rarity of this clinical scenario makes such studies challenging.

## Conclusions

Anterior-only rigid plate fixation appears to be a safe and effective strategy for traumatic sternal fractures crossing a previous sternotomy in carefully selected patients. By avoiding retrosternal dissection, this approach eliminates the risk of cardiac injury from dense post-surgical adhesions while providing adequate biomechanical stability through monocortical locking screws. This case highlights anterior-only rigid plating as a practical concept for stabilizing complex sternal fractures traversing previous sternotomy without exposing the patient to the hazards of posterior dissection. A thorough review of prior cardiac surgical history should be integral to preoperative planning for any sternal fracture requiring fixation.

## Supplementary Information

Supplementary Material 1

## Data Availability

All data generated or analyzed during this study are included in this published article. No additional datasets were generated or analyzed.

## Abbreviations

AO/OTA: Arbeitsgemeinschaft für Osteosynthesefragen / Orthopaedic Trauma Association
CT: Computed tomography
